# Integrated Transcriptomic and Metabolomic Analysis Reveals the Mechanism of Gibberellic Acid Regulates the Growth and Flavonoid Synthesis in *Phellodendron chinense* Schneid Seedlings

**DOI:** 10.3390/ijms242216045

**Published:** 2023-11-07

**Authors:** Lv Yang, Shengwei Luo, Jing Jiao, Wende Yan, Baiquan Zeng, Hanjie He, Gongxiu He

**Affiliations:** 1 National Engineering Laboratory for Applied Technology of Forestry and Ecology in South China, Hunan Provincial Key Laboratory of Forestry Biotechnology, College of Life Sciences and Technology, Central South University of Forestry & Technology, Changsha 410004, China; lvyang213629@163.com (L.Y.); 13574832060@163.com (S.L.); jj15293490038@163.com (J.J.); csfuywd@hotmail.com (W.Y.); baiquanzhn@163.com (B.Z.); 2 College of Forestry, Central South University of Forestry & Technology, Changsha 410004, China

**Keywords:** *Phellodendron chinense* Schneid, geberellic acid, phytohormone signal transduction, flavonoid, transcriptomics, metabonomics

## Abstract

The phytohormone gibberellic acids (GAs) play a crucial role in the processes of growth, organ development, and secondary metabolism. However, the mechanism of exogenous GA_3_ regulating the growth and flavonoid synthesis in *Phellodendron chinense* Schneid (*P. chinense* Schneid) seedlings remains unclear. In this study, the physicochemical properties, gene expression level, and secondary metabolite of *P. chinense* Schneid seedlings under GA_3_ treatment were investigated. The results showed that GA_3_ significantly improved the plant height, ground diameter, fresh weight, chlorophyll content, soluble substance content, superoxide dismutase, and peroxidase activities. This was accompanied by elevated relative expression levels of *Pc(S)-GA2ox*, *Pc(S)-DELLA*, *Pc(S)-SAUR50*, *Pc(S)-PsaD*, *Pc(S)-Psb 27*, *Pc(S)-PGK*, *Pc(S)-CER3,* and *Pc(S)-FBA* unigenes. Conversely, a notable reduction was observed in the carotenoid content, catalase activity and the relative expression abundances of *Pc(S)-KAO, Pc(S)-GID1*/*2,* and *Pc(S)-GH 3.6* unigenes in leaves of *P. chinense* Schneid seedlings (*p* < 0.05). Furthermore, GA_3_ evidently decreased the contents of pinocembrin, pinobanksin, isosakuranetin, naringin, naringenin, (−)-epicatechin, tricetin, luteolin, and vitexin belonged to flavonoid in stem bark of *P. chinense* Schneid seedlings (*p* < 0.05). These results indicated that exogenous GA_3_ promoted growth through improving chlorophyll content and gene expression in photosynthesis and phytohormone signal pathway and inhibited flavonoid synthesis in *P. chinense* Schneid seedlings.

## 1. Introduction

Plant growth and secondary metabolism are influenced by various plant growth regulators, including abscisic acid (ABA), auxin, and gibberellic acids (GAs) [1,2]. GA_3_ is characterized as a tetracyclic diterpenoid compound that fulfills vital functions in numerous plant processes, encompassing seed germination, stem elongation, flowering, gene expression, signal transduction, and secondary metabolism in plants [3,4,5,6]. Previous studies have demonstrated that exogenous GA_3_ significantly improves the photosynthetic pigment content, photosynthetic efficiency, soluble substance content, and antioxidase activities. Concurrently, it increases the fresh weight and plant height, subsequently promoting plant growth [2,7,8,9,10,11].

Exogenous GA_3_ regulates the biosynthesis and its signal transduction pathway of endogenous phytohormone, which controls the plant growth [12,13]. The appropriate concentration of exogenous GA_3_ influences the endogenous levels of phytohormones, such as GA, ABA, and auxin, which, in turn, regulates various aspects of plant growth and development. The synthesis and signal transduction of these phytohormones are controlled by specific genes, such as *GA20 OXIDASE (GA20ox*), *PYR/PYL*, and *Gretchen Hagen 3* (*GH3*) [14,15,16]. In previous studies, it was demonstrated that exogenous GA_3_ reduces the relative expression levels of *ZmGA20 OXIDASE7* (*GA20ox7*) and *ZmGA3 OXIDASE1/3* (*GA3ox1/3*) genes, and enhances the levels of *ZmGA2 OXIDASE1/4* (*GA2ox1/4*) and *ZmGA20 OXIDASE2* (*GA20ox2*) genes in GA biosynthesis in maize [17]. Meanwhile, exogenous GA_3_ elevates the relative expression levels of major genes in GA signal pathway while decreasing the levels of *NcGA20ox* and *NcGA3ox* genes in biosynthesis after 3 days, ultimately promoting the growth of *Neolamarckia cadamba* [7]. In addition, exogenous GA_3_ impacts the biosynthesis and accumulation of other phytohormones, such as ABA, auxin, and cytokinin (CTKs), to balance the plant growth [12,18].

Secondary metabolites comprise a diverse group of compounds, including flavonoids, alkaloids, and terpenoids, where flavonoids play a protective role against biotic and abiotic stresses. The biosynthesis of these compounds is regulated by exogenous GA_3_ in plants [19,20]. Previous studies have validated that exogenous GA_3_ inhibited the biosynthesis of flavonoid and isoflavonoid through negatively regulating GA-mediated signal pathway and promoted the growth by improving nitrogen metabolism in *Medicago truncatula* [21]. The flavonoid biosynthesis is a sophisticated process, and the application of exogenous GA_3_ has been shown to decrease their content. This occurs through the downregulation of the relative expression levels of key genes involved in flavonoid biosynthesis and the suppression of enzyme activities [22,23,24]. Furthermore, exogenous GA_3_ reduces the flavonoid content by inhibiting the expression levels of certain transcription factors related to flavonoid biosynthesis. Alternatively, it may indirectly depress the biosynthesis of endogenous phytohormones, ultimately promoting flavonoid accumulation [22,25].

*Phellodendron chinense* Schneid (*P. chinense* Schneid) is a traditional woody medicinal plant in China that mainly contains alkaloid, flavonoid, and terpenoid compounds in stem bark [26]. These active ingredients possess significant antibacterial and anti-inflammatory, antihypertensive, and lipid-lowering effects. They also demonstrate efficacy in inhibiting tumor proliferation and enhancing immunity. As a result, they find widespread application in the treatment of conditions such as hypertension, enteritis, and other diseases [27,28,29]. The content of active components in *P. chinense* Schneid determines the quality and benefit, and its biosynthesis and accumulation are affected by exogenous GA_3_. In a previous study, we demonstrated that exogenous GA_3_ (50 mg, 100 mg, and 200 mg L^−1^) treatment promotes the growth of *P. chinense* Schneid seedlings, and the promotion of 100 mg L^−1^ concentration was better compared with the control group (CK) (Table S1). However, the molecular mechanism of exogenous GA_3_ regulating the growth and secondary metabolism in *P. chinense* Schneid seedlings remains unclear. Therefore, this study aimed to investigate the effect of exogenous GA_3_ on the physicochemical properties, differentially expressed genes (DEGs), and differentially accumulated metabolites (DAMs) in leaves and stem bark and revealed the molecular mechanism of exogenous GA_3_ regulating the growth and flavonoid synthesis in *P. chinense* Schneid. These findings may help to provide a theoretical basis for the scientific planting and quality improvement of *P. chinense* Schneid.

## 2. Results

### 2.1. GA_3_ Promotes Plant Growth

**Figure 1:**
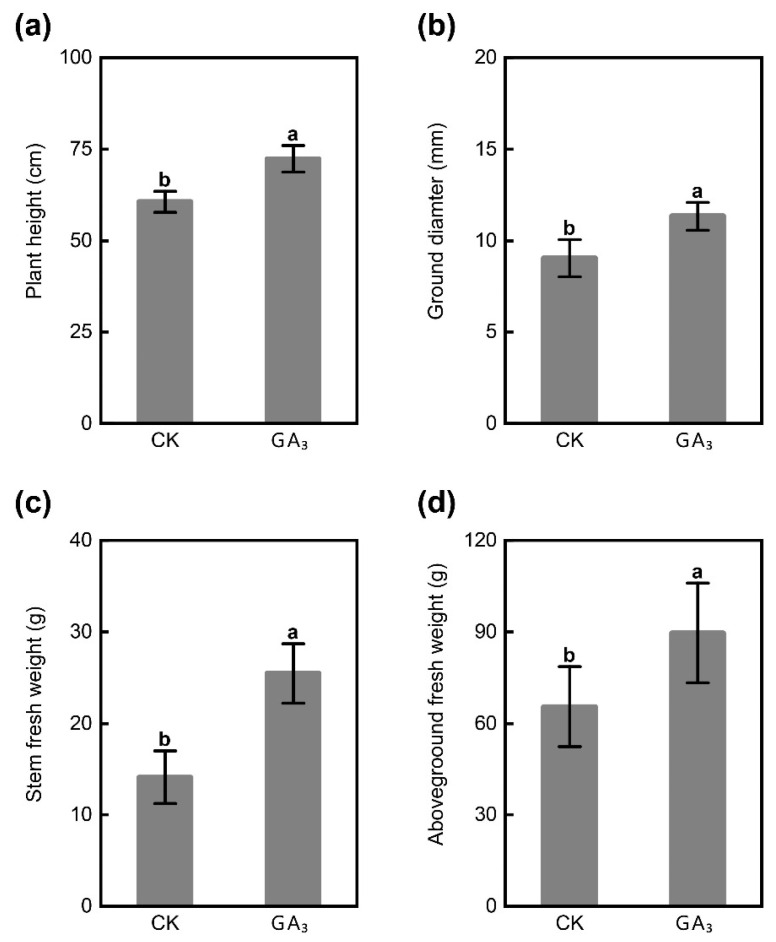
Effect of exogenous GA_3_ on the growth of *P. chinense* Schneid seedlings. (**a**) The plant height. (**b**) The ground diameter. (**c**) The stem fresh weight. (**d**) The aboveground fresh weight. All values are presented as means ± SD of three independent experiments (*n* = 3). The different letters on the bars of the same parameter indicate significant difference (*p* < 0.05).

The application of GA_3_ led to significant enhancements in both plant height and ground diameter of *P. chinense* Schneid seedlings, showing an increase of approximately 1.19- and 1.25-fold, respectively, compared to the control group (CK) (Figure 1a,b). Under the same condition, the stem fresh weight and aboveground fresh weight in GA_3_ treatment group were also increased, with 1.81- and 1.37-fold compared with that in CK treatment, and possessed a distinct difference (Figure 1c,d). These results indicated that exogenous GA_3_ observably promoted the growth of *P. chinense* Schneid seedlings.

### 2.2. GA_3_ Regulates Physicochemical Property

**Figure 2:**
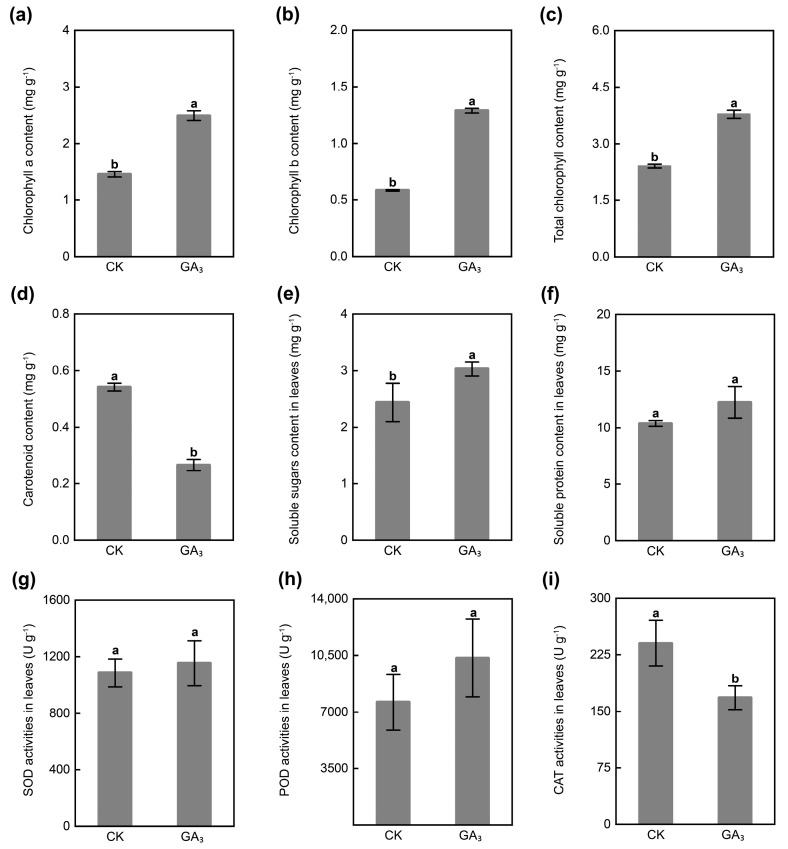
Effect of exogenous GA_3_ on the physicochemical property in leaves of *P. chinense* Schneid seedlings. (**a**) The chlorophyll a content. (**b**) The chlorophyll b content. (**c**) The total chlorophyll content. (**d**) The carotenoid content. (**e**) The soluble sugar content. (**f**) The soluble protein content. (**g**) The SOD activity. (**h**) The POD activity. (**i**) The CAT activity. All values are presented as means ± SD of three independent experiments (*n* = 3). The different letters on the bars of the same parameter indicate significant difference (*p* < 0.05).

The application of GA_3_ significantly increased the contents of chlorophyll a (Chl a), chlorophyll b (Chl b), and total chlorophyll (Chl) in leaves, with 1.72-, 2.20-, and 1.57-fold, while it observably decreased the carotenoid content in leaves, with 0.49-fold compared with that in CK group (Figure 2a–d). At the same time, GA_3_ enhanced the contents of soluble sugar and soluble protein in leaves, with 1.24- and 1.18-fold compared to the CK group (Figure 2e,f). These results indicated that exogenous GA_3_ significantly improved the contents of Chl and soluble sugar in the leaves of *P. chinense* Schneid seedlings.

Compared with CK, GA_3_ treatment improved the activities of superoxide dismutase (SOD) and peroxidase (POD) in leaves, with 1.06-fold and 1.36-fold, whereas it remarkably reduced the catalase (CAT) activity and malondialdehyde (MDA) content in leaves, with 0.70-fold and 0.41-fold (Figure 2g–i and Figure S1). These results demonstrated that exogenous GA_3_ improved the activities of SOD and POD in the leaves of *P. chinense* Schneid seedlings.

### 2.3. Correlation Analysis of Growth Index with Physiological Index

The plant height showed a significant negative correlation with MDA and carotenoid, while it had a distinct positive correlation with Chl a, Chl b, and total Chl. The ground diameter presented a significant negative correlation with carotenoid, whereas it had a distinguishable positive correlation with Chl b and total Chl. The stem fresh weight possessed a notable negative correlation with MDA and carotenoid and it showed a distinct positive correlation with Chl a, Chl b, total Chl, and soluble protein. The aboveground fresh weight was significantly correlated with stem fresh weight and ground diameter (Figure S2a). The results of the Random Forest Test showed that the total Chl was the main factor for promoting the growth of *P. chinense* Schneid seedlings (Figure S2b).

### 2.4. Transcriptome Analysis in Leaves

In this present study, a total of 6 RNA libraries were prepared and analyzed using leaves from *P. chinense* Schneid seedlings subject to GA_3_ and normal condition treatments. After removing the reads with linkers and low-quality reads, 20.89~21.78 million clean reads (6.25~6.52 Gb) were obtained, the G-C contents ranged from 43.41% to 44.68%, and Q30 ranged from 92.25% to 93.29% (Table 1). A total of 442,227 transcripts (726.71 Mb in size) were obtained, with a mean length of 1643.30 bp and an N50 length of 2436 bp. Subsequently, de novo assembly of the clean reads produced 101,559 unigenes (73.92 Mb in size) with a mean length of 727.89 bp and an N50 length of 1431 bp. Furthermore, a total of 9216 unigenes (9.07%) and 145,775 transcripts (32.96%) were longer than 2000 bp (Table S2). The unigenes obtained from assembly were annotated by BLAST (E-value ≤ 1 × 10^−5^) alignment in eight public databases (Clusters of Orthologous Groups Database, COG; Gene Ontology Database, GO; Kyoto Encyclopedia of Genes and Genomes Database, KEGG; Clusters of Eukaryotic Orthologous Groups, KOG; the Protein Family Database, Pfam; Swiss-Prot Protein Sequence Database, Swissprot; Evolutionary genealogy of genes: Non-supervised Orthologous Database, eggNOG and Non-Redundant Protein Sequence Database, Nr). A total of 13,574 (5.88%), 31,747 (13.75%), 17,745 (7.69%), 25,104 (10.88%), 26,407 (11.44%), 27,444 (11.89%), 41,511 (17.98%), and 47,307 (20.49%) unigenes were annotated in the COG, GO, KEGG, KOG, Pfam, Swissprot, eggNOG, and Nr databases (Table S3). 

The KEGG database was used to identify 21 important pathways involved in various developmental processes with a total of 19,060 unigenes in plants (Figure S3a), among which the “Metabolism (12,367)”, “Genetic information processing (4438)”, “Environmental information processing (539)”, “Cellular process (1349)”, “Organismal system (336)”, and “Human diseases” (31) were the primary terms. A total of 21 important pathways were identified, encompassing a range of developmental and metabolic processes in leaves were identified, among which the most prominent pathways were “carbohydrate metabolism (3348)”, “translation (2163)”, and “transport and catabolism (1349)”.

GO terms associated with each unigene were identified based on sequence homology (Figure S3b). A total of 31,747 unigenes were classified into 50 GO terms which were assigned to three dominant categories of biological processes (BPs), cellular components (CCs), and molecular functions (MFs). In BP category, the unigenes were further divided into 20 terms, among which “metabolic process (17,031)”, “cellular process (15,023)”, and “single-organism process (10,707)” represented the dominant terms. In the CC category, the unigenes were further divided into 15 terms, and the greatest numbers of unigenes (14,034, 13,956, and 10,072) were annotated to “cell”, “cell part”, and “organelle”. In MF category, the unigenes were further divided into 15 terms, among which the “binding (16,060)”, “catalytic activity (15,972)”, and “transport activity (2150)” were the main terms.

### 2.5. Differentially Expressed Genes (DEGs) Analysis

**Figure 3:**
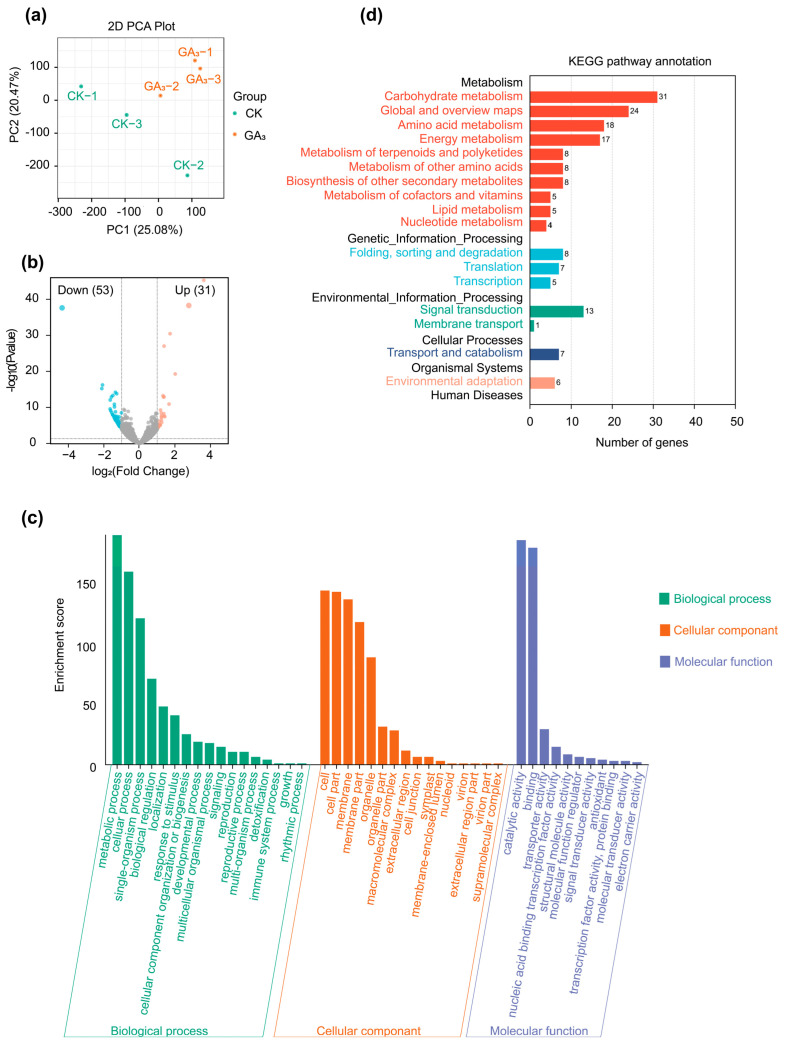
Transcriptomic analysis of leaves in *P. chinense* Schneid seedlings under exogenous GA_3_ treatment. (**a**) PCA analysis of transcriptome data. (**b**) Volcano map of DEGs in exogenous GA_3_ treatment sample. (**c**) GO enrichment analysis of DEGs. (**d**) KEGG enrichment analysis of DEGs.

The result of principal component analysis (PCA) showed that samples of GA_3_ treatment were significantly separated from the CK experimental group, indicating that exogenous GA_3_ had a distinct influence on leaves of *P. chinense* Schneid seedlings and could perform the next analysis (Figure 3a). The DEGs were conducted between GA_3_ treatment and CK group in leaves. A total of 570 DEGs were identified, among which 266 DEGs were upregulated and 304 DEGs were downregulated (*p* < 0.01, Fold Change ≥ 2) (Figure 3b). GO enrichment revealed that 570 DEGs were significantly enriched in metabolic processes (Biological process), cell (Cellular component), and catalytic activity (Molecular function) (Figure 3c). Furthermore, among 570 DEGs in GA_3_ treatment group, 173 DEGs were annotated to the top 17 pathways in KEGG database, among which the “metabolism (128)”, “genetic information processing (20)”, and “environmental information processing (14)” were the primary terms, the “Plant hormone signal transduction (six upregulated and seven downregulated)”, “carbohydrate metabolism (five upregulated and seven downregulated)”, and “amino acid biosynthesis (five upregulated and three downregulated)” were the main pathways. In the plant hormone signal pathways, the GA signal pathway had five DEGs (two upregulated and three downregulated), the IAA signal pathway contained four DEGs (two upregulated and two downregulated), the CTK signal pathway included one upregulated DEGs, the ABA signal pathway had two DEGs (one upregulated and one downregulated). Furthermore, the photosynthetic system contained four DEGs (three upregulated and one downregulated), and the carbon metabolism had nine DEGs (four upregulated and five downregulated) (Figure 3d). 

### 2.6. Analysis of DEGs during Photosynthesis and Carbon Metabolism

**Figure 4:**
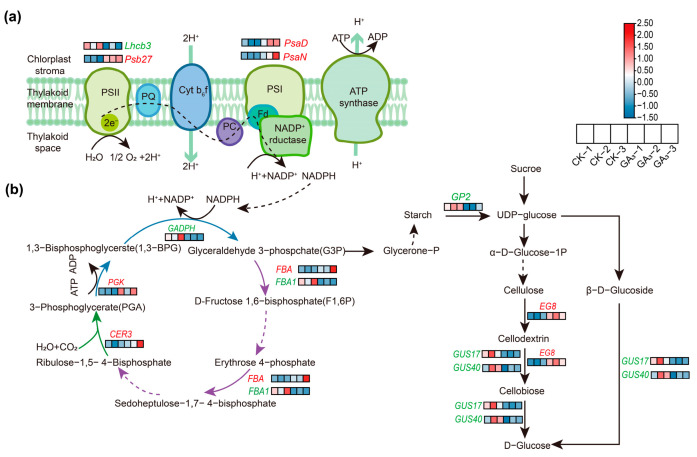
Analysis of DEGs involved in photosynthesis and carbon metabolism in leaves of *P. chinense* Schneid seedlings under exogenous GA_3_ treatment. (**a**) DEGs involved in photosynthesis. (**b**) DEGs during carbon metabolism. Red colors represent upregulation, and green colors represent downregulation. Dashed lines represent the two-step and multi-step reactions.

In the photosynthetic system, a total of four DEGs in leaves under GA_3_ treatment were identified, among which the *Pc(S)-Psb27* (*PHOTOSYSTEM II Psb27*, c52610.graph_c0), *Pc(S)-PsaD* (*PHOTOSYSTEM I SUBUNIT II*, c49896.graph_c0) and *Pc(S)-PsaN* (*PHOTOSYSTEM I SUBUNIT PsaN*, c56131.graph_c0) unigenes were upregulated, and the *Pc(S)-Lhcb3* (*LIGHT-HARVESTING COMPLEX II CHLOROPHYLL a/b BINDING PROTEIN*, c59603.graph_c0) unigene was downregulated (Figure 4a). 

Within carbon metabolism, a total of five DEGs were identified in experimental samples between GA_3_ treatment and CK groups, among which the *Pc(S)-CER3* (*CERIFERUM 3*, c57178.graph_c0), *Pc(S)-PGK* (*PHOSPHOGLYCERATE KINASE*, c61467.graph_c0) and *Pc(S)-FBA* (*FRUCTOSE-BISPHOSPHATE ALDOLASE*, c56524.graph_c0) unigenes were upregulated, whereas the *Pc(S)-FBA1* (c63247.graph_c0) and *Pc(S)-GAPDH* (*GLYCERALDEHYDE-3-PHOSPHATE DEHYDROGENASE*, c65707.graph_c0) unigenes were downregulated. In the sugar metabolism pathway, the *Pc(S)-GUS17/40* (*β-GLUCOSIDASE17/40*, c62209.graph_c0 and c66754.graph_c0) and *Pc(S)-GP2* (*α-GLUCAN PHOSPHORYLASE2*, c64150.graph_c0) unigenes were downregulated, whereas the *Pc(S)-EG8* (*ENDOGLUCANASE8*, c63908.graph_c0) unigene was upregulated (Figure 4b).

### 2.7. Analysis of DEGs during Hormone Signal Pathway

**Figure 5:**
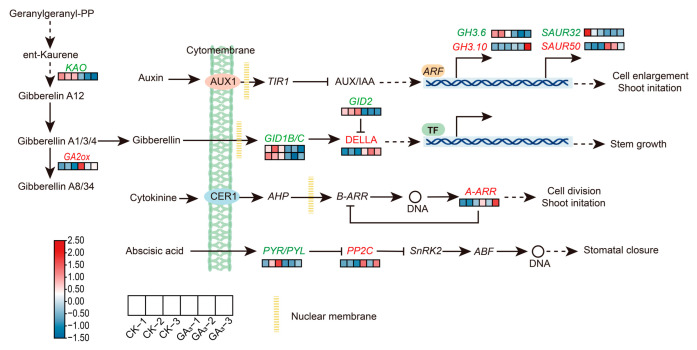
Analysis of DEGs in phytohormone signal pathways. Red colors represent upregulation, and green colors represent downregulation. Dashed lines represent the two-step and multi-step reactions.

In the GA signal pathway, a total of six DEGs (two upregulated and four downregulated) in GA_3_ treatment sample were identified, among which the *Pc(S)-KAO* (c65280.graph_c0), *Pc(S)-GID1B/C* (c66756.graph_c0, c69102.graph_c0), and *Pc(S)-GID2* (c56133.graph_c0) unigenes were downregulated, whereas the *Pc(S)-GA2ox* (c56918.graph_c0) and *Pc(S)-DELLA* (c24239.graph_c0) unigenes were upregulated compared with that in CK group, indicating that exogenous GA_3_ inhibited biosynthesis and signal transduction of GA hormone in leaves of *P. chinense* Schneid seedlings (Figure 5). Under the same condition, a total of four DEGs (two upregulated and two downregulated) were identified in auxin signal pathway, among which the *Pc(S)-SAUR50* (c55986.graph_c1) and *Pc(S)-GH3.10* (c43034.graph_c0) unigenes were upregulated, while the *Pc(S)-SAUR32* (c24304.graph_c0) and *Pc(S)-GH3.6* (c69394.graph_c0) unigenes were downregulated, demonstrating that exogenous GA_3_ played a key role in IAA signal transduction (Figure 5).

In the ABA signal pathway, a total of 2 DEGs (1 upregulated and 1 downregulated) in leaves under GA_3_ treatment were identified, among which the *Pc(S)-PYR/PYL* (c60619.graph_c0) unigene was downregulated, and the *Pc(S)-PP2C* (c65623.graph_c0) unigene was upregulated compared with that in CK group, showing that exogenous GA_3_ inhibited ABA signal pathway in leaves of *P. chinense* Schneid seedlings. Moreover, the *Pc(S)-A-ARR* (c36315.graph_c0) involved in CTK signal pathway was upregulated in GA_3_ treatment sample, indicating that exogenous GA_3_ regulated CK signal pathway in the leaves of *P. chinense* Schneid seedlings (Figure 5).

### 2.8. Differentially Accumulated Metabolites (DAMs) Analysis

The differences in the metabolites in stem bark from CK and GA_3_ treatment samples were distinguishable by 2D PCA analysis. In the PCA score plot, two principal components (PC1 and PC2) were extracted to be 31.45% and 23.54%, and the three biological replicates of GA_3_ treatment group were significantly clustered, which obviously separated from CK group, indicating that the GA_3_ had an important impact on the metabolites in the stem bark of *P. chinense* Schneid seedlings (Figure S4a). Meanwhile, the cluster and class heatmaps showed the similarity of components among biological repeats and the difference of components among the two treatment groups, suggesting that GA_3_ strongly influenced the metabolite profiles in the stem bark of *P. chinense* Schneid seedlings (Figure S4b). In this present study, a total of 443 metabolites in stem bark samples from CK and GA_3_ treatment groups were identified and divided into 11 categories, which mainly included flavonoid (90), phenolic acid (62), lipid (59), amino acid and derivative (46), alkaloid (45), organic acid (31), nucleotide and derivative (30), and lignan and coumarin (20) (Figure S4c).

**Figure 6:**
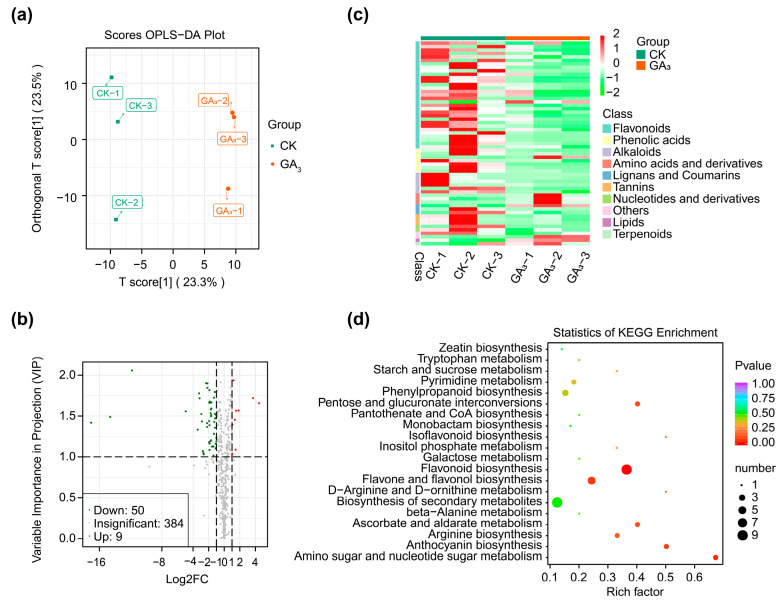
Metabolome analysis of stem bark in *P. chinense* Schneid seedlings under exogenous GA_3_ treatment. (**a**) OPLS-DA analysis of metabolome. (**b**) Volcano map of DAMs. (**c**) Heatmap analysis of clustered DAMs. (**d**) KEGG enrichment analysis of DEGs.

The Orthogonal Partial Least Squares-Discriminant Analysis (OPLS-DA) showed that two principal components (T score and orthogonal T score) were extracted to be 23.3% and 23.5%, and the three biological replicates of GA_3_ treatment group were clustered, which distinctly separated from CK group, demonstrating that this experiment was reproducible and reliable, and GA_3_ had an important effect on the DAMs in stem bark of *P. chinense* Schneid seedlings (Figure 6a). Based on FC ≥ 2 or ≤ 0.5 and VIP ≥ 1, the DAMs in stem bark from GA_3_ group were identified compared with CK in this study. A total of 59 DAMs (9 upregulated and 50 downregulated) were annotated in KEGG database, which primarily included flavonoid (1 upregulated and 30 downregulated), phenolic acid (one upregulated and six downregulated), alkaloid (six downregulated), lignan and coumarin (one upregulated and two downregulated), amino acid and derivative (three upregulated), nucleotide and derivative (two downregulated), terpenoid (one upregulated), and lipid (one upregulated) (Figure 6b–d). According to the variation multiple, the DAMs in the Top 14 were apigenin-7-O-β-D-glucuronide (pmn001697), vitexin (mws0048), tamarixetin (mws2627), isorhamnetin (mws0066), naringenin(pme0376), pinobanksin (mws0914), 7,8-dihydroxyrutaecarpine (pmp000519), N-cis-sinapoyltyramine (pmp001178), N-cis-feruloyltyramine (pmp001256), L-(+)-arginine (mws0260), H-homoarg-OH (pme3388), L-citrulline (pme0008), O-feruloyl coumarin (pmb0235) and 7-methoxycoumarin (mws1075), indicated that exogenous GA_3_ chiefly regulated the flavonoid biosynthesis in the stem bark of *P. chinense* Schneid seedlings (Figure S4d).

### 2.9. DAMs during Flavonoids Synthesis Pathway

**Figure 7:**
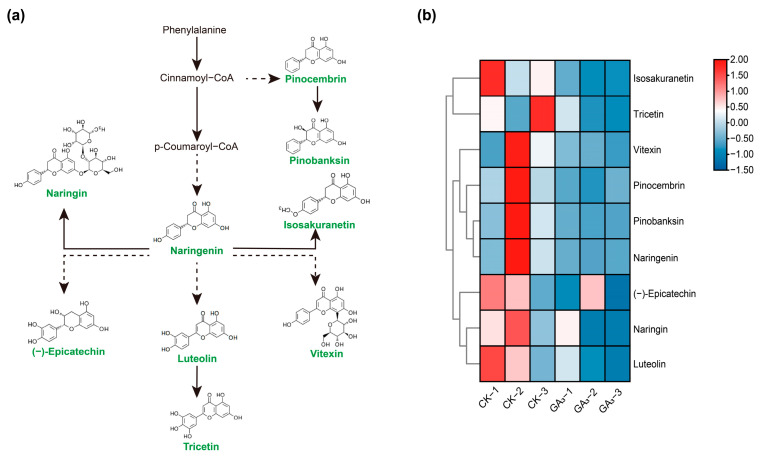
Analysis of DAMs in flavonoids biosynthesis in the stem bark of *P. chinense* Schneid seedlings under exogenous GA_3_ treatment. (**a**) The DAMs involved in flavonoid biosynthesis pathway. (**b**) Heatmap of DAMs in the flavonoid biosynthesis pathway. Green colors represent downregulation. Dashed lines represent the two-step and multi-step reactions.

The DAMs obtained from stem bark metabolites mainly enriched metabolic pathways (10), flavonoid biosynthesis (9), biosynthesis of secondary metabolites (9), flavone and flavonoid biosynthesis (5), and phenylpropanoid biosynthesis (3). In the flavonoid biosynthesis pathway, a total of 9 DAMs were identified, among which the pinocembrin (pmp000562), isosakuranetin (mws1034), naringin (mws0046), naringenin (pme0376), pinobanksin (mws0914), tricetin (pmb0713), luteolin (pmb0566), vitexin (mws0048), and (−)-epicatechin (pme0460) were all downregulated, indicating that exogenous GA_3_ inhibited the flavonoid biosynthesis in stem bark of *P. chinense* Schneid seedlings (Figure 7). 

## 3. Discussion

GA_3_ plays crucial roles in the processes of growth, seed germination, physicochemical properties, nutrient uptake, secondary metabolism, and stress response in plants [30,31]. The previous results showed that exogenous GA_3_ promoted the growth of *Neolamarckia cadamba* and *Camellia sinensis* seedlings and increased the biomass [7,32]. At the same time, GA_3_ enhanced the chlorophyll content but reduced carotenoid content in the leaves of *Valencia oranges*, suggesting that GA_3_ promoted growth by improving chlorophyll content in plants [33]. In the present study, GA_3_ application significantly improved chlorophyll content, increased plant height, ground diameter, and fresh weight, whereas it decreased carotenoid content in leaves, indicating that GA_3_ promoted growth through improving chlorophyll content in the leaves of *P. chinense* Schneid seedlings. The results were consistent with the findings of *Valencia oranges* and *Camellia sinensis* seedlings [33]. Under GA_3_ treatment, the increase in chlorophyll content in leaves may facilitate light absorption and carbon assimilation for improving photosynthetic efficiency, ultimately promoting the growth of *P. chinense* Schneid seedlings [34].

At the same time, GA_3_ treatment significantly increased the contents of soluble sugar and soluble protein, concurrently enhanced the activities of SOD and POD, but distinctly reduced MDA content and CAT activity in leaves, demonstrating that GA_3_ promoted the accumulation of soluble substance and improved antioxidant capacity of *P. chinense* Schneid seedlings, and were similar to the previous findings [10,35]. The GA_3_ enhances the antioxidase activity, which keeps the integrity of chloroplast structure for improving the photosynthetic efficiency in leaves, and then promotes the biosynthesis and accumulation of soluble sugar and soluble protein, providing precursor materials and energies for anabolism during the growth process. These results demonstrated that GA_3_ promoted the growth of *P. chinense* Schneid seedlings by improving chlorophyll content and antioxidant capacity.

The photosynthesis apparatus consists of two key components: photosystem I (PSI) and photosystem II (PSII) which are multisubunit pigment-protein complexes that catalyze the light-driven water oxidation, electron transport, energy conversion, and state transition, but these processes are controlled by genes and regulated by exogenous GA_3_ in plants [7,36,37,38,39]. The exogenous GA_3_ elevated the relative expression abundances of *Pt-TUA6* and *Pt-DRP4C* genes, encoding important components of the chlorophyll division ring in leaves, concurrently improving the photosynthesis capacity and biomass of *Populus tomentosa* seedlings [36]. In the present study, exogenous GA_3_ treatment significantly enhanced the relative expression levels of *Pc(S)-Psb27* and *Pc(S)-PsaD/N* unigenes in leaves, suggesting that exogenous GA_3_ promoted the growth through improving relative expression levels of the photosynthetic genes in leaves of *P. chinense* Schneid seedlings, these results were consistent with the chlorophyll contents and fresh weights. The Psb27 protein is a lumenal protein and auxiliary factor associated with PSII, which binds to the large extrinsic E-loop of CP43 at the lumenal side, maintains the stability of the D1 protein structure in PSII reaction center, and then improves the photosynthetic capacity and promotes plant growth [40,41,42]. The PsaD/N are crucial for PSI function. The former associates with PsaC/E polypeptides to form a stromal ridge that regulates the thioredoxin-mediated photoreduction of NADP^+^, the latter combines with PsaG/F polypeptides to form trimer that regulating the plastocyanin function, subsequently improving photosynthetic efficiency and biomass in plants [43,44,45,46]. Therefore, our results indicated that GA_3_ promoted growth by improving the relative expression levels of key genes in photosynthesis in the leaves of *P. chinense* Schneid seedlings.

Plant growth is a sophisticated process regulated by endogenous phytohormones, such as auxin, GA, ABA, and CTK, where biosynthesis is controlled by the exogenous GA_3_ [47,48,49]. The previous results showed that exogenous GA_3_ decreased the relative expression levels of *Nc-GA20ox* and *Nc-GA3ox* genes but increased the expression abundances of key genes in the auxin pathway, thereby promoting the growth of *Neolamarckia cadamba* seedlings [7]. In the present study, GA_3_ evidently reduced the relative expression levels of *Pc(S)*-*KAO*, *Pc(S)-GA2ox,* and *Pc(S)-GID1/2* unigenes, while notably increasing the transcript level of *Pc(S)-DELLA* unigene in leaves of *P. chinense* Schneid seedlings. The *Pc(S)*-*KAO* unigene participates in GA biosynthesis. The *Pc(S)-GA2ox* unigene is involved in the conversion of its inactive form, indicating that GA_3_ prevented GA biosynthesis and promoted its degradation in leaves of *P. chinense* Schneid seedlings [49,50]. The GID1B/C proteins as receptors recognized GA_3_ that combined with DELLA protein to form a trimer, subsequently labeled by SCF^SLY1^/GID2 complex and degraded through 26S protease, releasing the repression of DELLA protein and promoting cell elongation in plants [51,52]. Thus, the expression variations of *Pc(S)-GID1/2* (downregulation) and *Pc(S)-DELLA* (upregulation) unigenes demonstrated that exogenous GA_3_ inhibited the GA signal transduction in leaves of *P. chinense* Schneid seedlings, speculating that GA_3_ may promote its growth by other phytohormones. 

In addition, GA_3_ treatment reduced the relative expression levels of *Pc(S)-GH3.6* and *Pc(S)-SAUR32* unigenes but elevated the transcript levels of *Pc(S)-GH3.10* and *Pc(S)-SAUR50* unigenes in auxin signal pathway in leaves of *P. chinense* Schneid seedlings. The GH3 acyl acid amido synthetase catalyzed the conjugation of auxin with amino acid that inhibited cell division and expansion and played a positive role in the processes of JA synthesis and its signal pathway, which, in turn, maintained the phytohormone homeostasis and regulated the growth and development in plants [53,54,55]. Thus, the downregulation of *Pc(S)-GH3.6* unigene facilitated the accumulation of free auxin in leaves, combined with *Pc(S)-GH3.10* unigene to maintain its homeostasis and promoted the growth of *P. chinense* Schneid seedlings. In the auxin signal pathway, the *SAUR32* as a response factor negatively influenced regulation during plant growth and development, but the *SAUR50* was responsible for hypocotyl elongation, cotyledon expansion, and leaf development by activating plasma membrane H^+^-ATPase, resulting in cell wall acidification in plants [56,57,58,59]. Consequently, the downregulation of *Pc(S)-SAUR32* unigene alleviated the inhibition of cell division and elongation, while the upregulation of *Pc(S)-SAUR50* unigene promoted the growth and development by improving cell wall acidification, demonstrating that GA_3_ promoted the growth of *P. chinense* Schneid seedlings by regulating key gene expressions of auxin in its synthesis and signal pathway. These results were consistent with the findings of *Neolamarckia cadamba* seedlings [7]. Furthermore, GA_3_ reduced the relative expression levels of *Pc(S)-PYR/PYL* unigene, whereas it increased the transcript levels of *Pc(S)-PP2C* and *Pc(S)-A-ARR* unigenes that played a negative role in CTK and ABA pathways, illuminated that GA_3_ repressed these signal pathways in leaves of *P. chinense* Schneid seedlings [60,61,62]. Therefore, our results indicated that GA_3_ promoted growth through regulating phytohormone homeostasis and auxin signal pathway in leaves of *P. chinense* Schneid seedlings.

GA_3_ not only promotes growth, but also influences the biosynthesis and accumulation of secondary metabolites in plants [48,63,64]. Appropriate concentration GA_3_ significantly increased the contents of total anthocyanin and artemisinin in *Vitis vinifera* (Syrah grapevine) and *Artemisia annua*, but decreased the flavonoid content in *Medicago truncatula*, showing that the GA_3_ had a distinct effect on secondary metabolites in different plants [21,64,65]. In the present study, the GA_3_ significantly reduced the flavonoid contents in stem bark, including pinocembrin, isosakuranetin, naringin, naringenin, pinobanksin, tricetin, luteolin, vitexin, and (−)-epicatechin, indicating that GA_3_ blocked the flavonoid biosynthesis of *P. chinense* Schneid seedlings. These results were consistent with the findings of *Medicago truncatula* [21]. In plants, the biosynthesis and accumulation of secondary metabolites are controlled by crucial genes and phytohormones, which are influenced by exogenous GA_3_, but GA_3_ usually plays a negative role in these processes [66]. Moreover, the correlation network analysis showed that *PsaD*, *Psb27*, *PsaN*, *DELLA*, *GA2ox*, *SAUR50*, *GH3.10*, *A-ARR*, *FBA, CER3*, *PGK,* and *EG8* genes showed a significant positive correlation with the growth of *P. chinense* Schneid seedlings. However, naringin, pinobanksin, naringenin, and pinocembrin showed a significant negative correlation with the growth of *P. chinense* Schneid seedlings. Thus, our results suggested that GA_3_ decreased flavonoid content in stem bark by repressing the expression levels of key genes associated with biosynthesis, which also illuminated a competitive inhibition between the growth and flavonoid biosynthesis of *P. chinense* Schneid seedlings.

**Figure 8:**
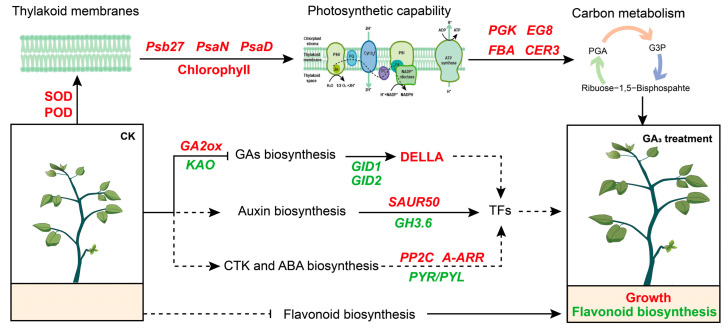
Theoretical mechanism of *P. chinense* Schneid seedlings response to exogenous GA_3_. Red colors represent upregulation, and green colors represent downregulation.

Based on the obtained results, a conceptual model was developed to describe the mechanism of exogenous GA_3_ regulating the growth and flavonoid biosynthesis of *P. chinense* Schneid seedlings (Figure 8). Briefly, exogenous GA_3_ enhanced the antioxidase (SOD and POD) activity and chlorophyll content that independently or cooperatively regulated key unigenes expression for improving photosynthetic capacity and carbon metabolism, subsequently promoting the growth of *P. chinense* Schneid seedlings. At the same time, GA_3_ mainly regulated the unigene expression in the signal pathways of GA, auxin, and ABA that synergistically promoted plant growth. On the other hand, exogenous GA_3_ inhibited flavonoid biosynthesis to alleviate the interference effect on the growth of *P. chinense* Schneid seedlings. However, the molecular mechanism of exogenous GA_3_ promoting growth through inhibiting flavonoid biosynthesis will require further investigation. 

## 4. Materials and Methods

### 4.1. Seedling Culture and Treatment

The study was conducted in a greenhouse at Central South University of Forestry and Technology, Changsha City, Hunan Province, China (28.1319° N, 112.9902° E). The mellow fruits of *P. chinense* Schneid were collected from Xiangxi Autonomous Prefector (Hunan Province), then seeds were separated and dried at room temperature. The seeds were placed in seedling discs containing peat substrate (Jiffy, Antwerp, Belgium) for germination and transferred to an experimental field (the soil was laterite, row spacing: 1.0 × 1.0 m) when the seedling height was 20 cm. When the height reached 30 cm, these seedlings were sprayed using a 100 mg L^−1^ GA_3_ solution three times at 5:00~5:30 p.m. on 0, 10, and 20 days, and the distilled water treatment served as a control group (CK) [32]. Each experimental group included six seedlings, which were collected 30 days after GA_3_ treatment, among which three seedlings were used for transcriptomic and metabolomic analysis, and the remaining seedlings were frozen in liquid nitrogen and stored at −80 °C for physicochemical property analysis. 

### 4.2. Growth Index Determination

The plant heights of collected seedlings were measured using a tape measure. The ground diameters were measured using a sliding caliper. The aboveground tissue and root of the collected seedlings were separated with scissors and then determined the aboveground fresh weights and the stem fresh weights which removed leaves from aboveground tissues with an analytical balance. Each experiment was repeated three times (*n* = 3).

### 4.3. Pigment Content Measurement

The pigment contents in the leaves of collected seedlings were measured according to the published literature [67]. Briefly, fresh leaves (0.2 g) of *P. chinense* Schneid seedlings were loaded into tubes that contained 10 mL 95% ethanol solution and placed in the dark condition for 24 h after sealing, then centrifuged at 11,000 rpm for 5 min. The total volumes of the extract solution were adjusted to 10 mL with 95% ethanol solution and then mixed well. The absorbances of extract solution at 470, 649, and 665 nm were detected using a spectrophotometer (UV754N, Yoke, Shanghai, China), and the pigment contents (mg g^−1^) in leaves of *P. chinense* Schneid seedlings were calculated. Each experiment was repeated three times (*n* = 3).

### 4.4. Soluble Sugar and Protein Content Determination

The soluble sugar contents in the leaves of collected seedlings were measured according to the published literature [68]. Briefly, fresh leaves (0.1 g) were ground in a mortar containing 5 mL of 80% ethanol solution, and the grinding fluid was moved into a 10 mL centrifuge tube in an 85 °C water bath for 30 min. After cooling to room temperature and centrifugation at 3000 rpm for 10 min, the supernatant was collected. The precipitate was extracted twice with 10 mL of 80% ethanol, and the supernatant was combined and fixed to 25 mL. The mixture (2 mL supernatant and 5 mL 2 g L^−1^ anthrone sulfate solution) was reacted at 100 °C for 10 min. The soluble sugar content was measured at 625 nm after cooling to room temperature. Each experiment was repeated three times.

Fresh leaves (0.5 g) were homogenized in a mortar containing 5 mL of 0.05 mol L^−1^ phosphate buffer (pH 7.8), the grinding fluid was moved into a 10 mL centrifuge tube, centrifuged at 4000 rpm for 10 min, and the supernatant was collected. Next 1 mL of extract and 5 mL of Coomassie brilliant blue G250 solution were mixed and left to rest for 2 min. Soluble protein content was measured at 595 nm. Each experiment was repeated three times.

### 4.5. Antioxidase Activity Detection

The fresh leaves (0.2 g) were milled into pulp in precooled mortar containing precooled 5 mL 0.05 M phosphate buffer (harbored 0.2 M disodium hydrogen phosphate and 0.2 M sodium dihydrogen phosphate), then the pulp was transferred into a centrifuge tube and centrifuged at 4 °C, 11,000 rpm for 20 min, subsequently, the supernatant was collected for measuring the antioxidase activity. The activities of superoxide dismutase (SOD) and catalase (CAT) in leaves were determined according to the published literature [69]. The SOD reaction mixture contained 50 mM phosphate buffer (pH 7.8), 13 mM methionine, 75 mM nitro blue tetrazolium chloride (NBT), 300 μM ethylenediaminetetraacetic acid (EDTA), 2 mM riboflavin, and 0, 50, 100, 150, and 200 μM enzyme extract. One unit of SOD activity was defined as the amount of enzyme required to cause 50% inhibition of NBT photoreduction. The CAT reaction mixture contained 50 mM phosphate buffer (pH 7.0), 10 mM H_2_O_2_, and enzyme extract. The specific activity of the enzyme was expressed in terms of micromolar H_2_O_2_ in min per mg of soluble protein. Peroxidase (POD) activity was determined following the published literature [70]. The absorbance of the reaction mixture containing 100 μL enzyme extract, 50 mM phosphate buffer (pH 7.0), 28 μL guaiacol, and 19 μL H_2_O_2_ was read at 420 nm with a 30 s interval up to 2 min and used the absorbance change 0.01 as a POD activity. Each experiment was repeated three times (*n* = 3).

### 4.6. Transcriptome Sequencing and Quality Control

Three independent biological replicates of leaves in CK and GA_3_ treatment group were used to extract total RNA with an RNAprep Pure plant kit (DP411, Tiangen, Beijing, China). The total RNA of each sample was quantified and qualified via Bioanalyzer (2100 Bioanalyzer, Agilent, CA, USA) and spectrophotometer (NanoDrop 2000, Thermo Fisher, Waltham, MA, USA). Thereafter, a total of 3 μg RNA from each sample was used as the input material for RNA sample preparation. Sequencing libraries were generated using the RNA-seq library Prep Kit for Illumina (VAHTS^®^ Universal V6, NEB, Ipswich, MA, USA). The generated sequencing libraries were qualified using a Qsep400 analyzer (Qsep400, Bioptic, Changzhou, China), then the eligible libraries were sequenced on Illumina platform (NovaSeq 6000, Illumina, CA, USA), and paired-end reads were generated. Subsequently, clean data (clean reads) were obtained by removing reads containing adapters, reads including poly-N sequences, and low-quality reads (Sanger base quality < 20) from raw data. Furthermore, the Q20, Q30, G-C contents and sequence duplication levels were calculated from the clean data. All downstream analyses were based on high-quality, clean data. 

### 4.7. Transcriptome Data Analysis

The clean reads were de novo assembled using Trinity software (V2.6.0, Broad Institute, Cambridge, MA, USA). Gene functions were annotated based on the following databases: Clusters of Orthologous Groups (COG), Gene Ontology (GO), Kyoto Encyclopedia of Genes and Genomes (KEGG), EuKaryotic Orthologous Groups (KOG), Protein family (Pfam), Swiss-Prot (a manually annotated and reviewed protein sequence database), evolutionary genealogy of genes: Non-supervised Orthologous Groups (eggNOG), and National Center for Biotechnology Information Non-Redundant protein sequences (Nr). Different expression analysis between CK and GA_3_ treatments was performed using the DESeq2 software. The obtained *p*-value was adjusted using Benjamini–Hochberg method [71] to control the false discovery rate (FDR). The FDR < 0.05 and log2 fold-change (|log2 Fold Change|) ≥ 2 were set as the threshold for screening significantly different expressions. 

### 4.8. Gene Annotation and Pathway Analysis

The GO term annotation of the DEGs was produced according to biological processes (BPs), cellular components (CCs), and molecular functions (MFs) through Blast2GO software (V2.5, Biobam, Valencia, Spain). The KEGG annotation of DEGs was conducted according to cellular processes, environmental information processing, genetic information processing, and metabolism and organismal systems. KOBAS software (V3.0, PKU, Beijing, China) was used to test the statistical enrichment of the DEGs in KEGG pathways. 

### 4.9. Metabolite Analysis

Each sample was placed in a lyophilizer (Scientz-100F, SCIENTZ, Ningbo, China) for vacuum freeze-drying and ground into powder (30 Hz, 1.5 min) using a grinder (MM 400, Retsch, Shanghai, China). Weigh 50 mg powder of each sample, add 1200 μL of 70% methanol water internal standard extract precooled at −20 °C, and vortex every 30 min for 30 s 6 times. Finally, after centrifugation at 12,000 rpm for 3 min, the supernatant was absorbed, and the sample was filtered with a microporous filter (0.22 μm pore size) and stored in an injection vial for UPLC-MS/MS analysis.

The data acquisition instrument system mainly includes Ultra Performance Liquid Chromatography (UPLC) (ExionLC™ AD, SCIEX, Framingham, MA, USA) and Tandem mass spectrometry (MS/MS) (TripleTOF 6600 LC-MS/MS, SCIEX, Framingham, MA, USA). Liquid phase conditions mainly include: the chromatographic column: Agilent SB-C18 (2.1 mm 100 mm,1.8 µm, Agilent, USA); Mobile phase: Phase A is ultrapure water containing 0.1% formic acid, Phase B is a solution of acetonitrile containing 0.1% formic acid; Elution gradient: 0.00 min B phase ratio of 5%, B phase proportion increased linearly to 95% within 9.00 min, and was maintained at 95% of 1 min, 10.00–11.10 min, Phase B ratio decreased to 5%, and balanced at 5% to 14 min; Flow rate: 0.35 mL min^−1^; Column temperature is 40 °C; The injection volume is 2 μL.

The MS conditions mainly include: electrospray ion source (electrospray ionization, ESI) temperature 550°C; ion spray voltage (IS) 5500 V (positive ion mode)/−4500 V (negative ion mode); ion source gas I (GSI), gas II (GSII), and air curtain gas (CUR) were set to 50, 60, and 25 psi, respectively, and collision-induced ionization parameters were set to high. The QQQ scan uses the MRM (multiple reaction monitoring, MRM) mode and sets the collision gas (N_2_) to medium. The decluster voltages (Declustering potential, DP; collision energy, CE) of each MRM ion pair were accomplished by optimizing the DP and CE. A specific set of MRM ion pairs was monitored during each period based on the metabolites eluted in each period.

### 4.10. Statistical Analysis

The data were analyzed using SPSS software (V22.0, IBM, Amonk, NY, USA) with a significant difference level. The significant differences in growth index, photosynthetic pigment content, soluble substance content, antioxidase activity, and MDA content in the leaves of *P. chinense* Schneid seedlings under exogenous GA_3_ treatment were analyzed by the methods of one-way analysis of variance (ANOVA) with 95% and a post hoc Tukey’s test. Pearson correlation analysis and the *t*-test were performed to integrate and visualize transcriptomics and metabonomics data sets. These graphs and random forest analysis were conducted using the R package “random Forest ” (V3.5.1, Lucent, NJ, USA) and Origin software (2021 version, Origin Lab, MA, USA). Finally, DEGs and DAMs were annotated in the KEGG database for the enrichment of metabolic pathways and the mapping of related pathways.

## 5. Conclusions

Exogenous GA_3_ significantly enhanced the chlorophyll content, fresh weight, soluble substance content, SOD and POD activities, evidently reducing the carotenoid content and CAT activity, indicating that GA_3_ promoted the growth of *P. chinense* Schneid seedlings by improving the chlorophyll content and some antioxidase activities. At the same time, exogenous GA_3_ elevated the relative expression levels of *Pc(S)-Psb27*, *Pc(S)-PsaD/N*, *Pc(S)-DELLA*, *Pc(S)-GH3.10*, *Pc(S)-SAUR50*, *Pc(S)-PP2C,* and *Pc(S)-A-ARR* unigenes, concurrently reduced the relative expression abundances of *Pc(S)*-*KAO*, *Pc(S)-GA2ox*, *Pc(S)-GID1/2*, *Pc(S)-GH3.6*, *Pc(S)-SAUR32,* and *Pc(S)-PYR/PYL* unigenes in leaves, revealed that GA_3_ promoted the growth of *P. chinense* Schneid seedlings through improving the relative expression levels of key unigenes in photosynthetic system and GA and auxin signal pathways. Furthermore, exogenous GA_3_ observably decreased the flavonoid content in stem barks, demonstrating that GA_3_ inhibited the biosynthesis and accumulation of flavonoids in *P. chinense* Schneid seedlings. Our results revealed that exogenous GA_3_ promoted the growth and inhibited flavonoid synthesis in *P. chinense* Schneid seedlings by regulating the relative expression levels of unigenes in photosynthesis and phytohormone signal pathways.

## Figures and Tables

**Table 1:** Summary of transcriptome sequencing results after filtering.

Applications ^a^	Treatments	Total Clean Reads ^b^	Total Clean Bases (bp)	G-C (%) ^c^	Q30 (%) ^d^	Mapped Reads ^e^	Mapped Ratio ^f^ (%)
	CK-1	21,182,430	6,332,375,932	44.68	93.29	16,329,151	77.09
CK	CK-2	21,770,028	6,520,262,422	43.65	93.15	16,529,517	75.93
	CK-3	20,888,663	6,249,140,252	43.76	92.68	16,066,835	76.92
	GA_3_-1	21,782,716	6,513,190,704	43.41	93.29	16,702,799	76.68
GA_3_	GA_3_-2	20,993,405	6,287,678,294	43.70	92.25	15,738,212	74.97
	GA_3_-3	21,035,971	6,288,453,804	43.50	92.55	16,069,780	76.39

^a^ For each treatment, three independent biological replicates were collected and sequenced. ^b^ Total clean reads were obtained after removing the reads containing linkers and low-quality reads. ^c^ G-C content of the bases in total clean reads. ^d^ Q30, the percentage of bases with a Phred quality score greater than 30. ^e^ Mapped reads, total clean reads were mapped. ^f^ Mapped ratio, the percentage of mapped read in total clean reads.

## Data Availability

Data are available on request from the corresponding authors.

## Supplementary Materials

The following supporting information can be downloaded at: https://www.mdpi.com/1422-0067/24/22/16045/s1.
