# Supplementary material for: Integrated Transcriptomic and Metabolomic Analysis Reveals the Mechanism of Gibberellic Acid Regulates the Growth and Flavonoid Synthesis in Phellodendron chinense Schneid Seedlings
Source: Int J Mol Sci. 2023 Nov 7;24(22):16045. doi: 10.3390/ijms242216045 (PMC10671667; doi:10.3390/ijms242216045)
Supplement: Supplementary file 1 [file ijms-24-16045-s001.zip › supplementary Figures.pdf]

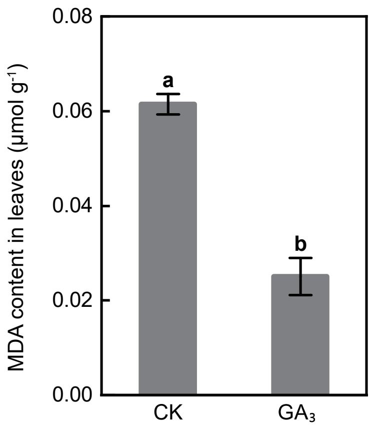

Figure S1: Effect of exogenous GA_3 on the MDA content in leaves of *P. chinense* Schneid seedlings. All values are presented as means ± SD of three independent experiments (n=3). The different letters on t-he bars of the same parameter indicate significant difference (P < 0.05).

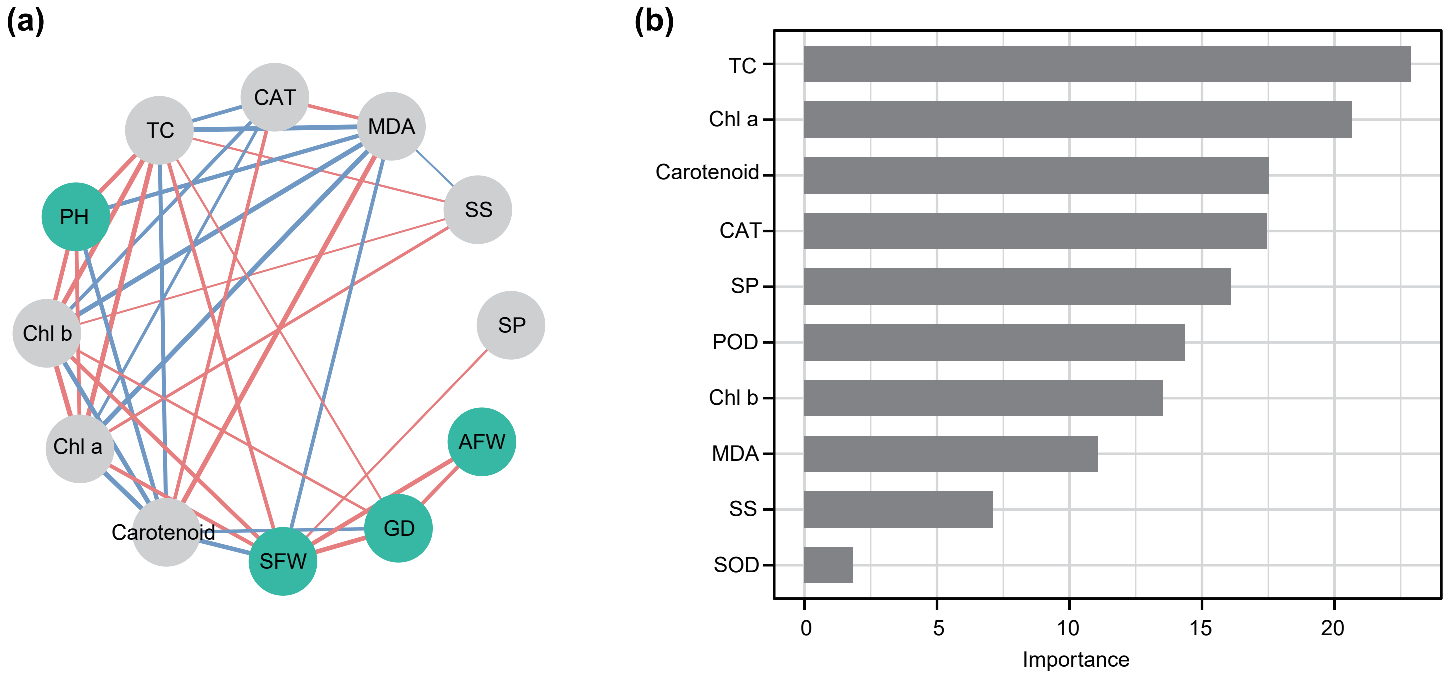

Figure S2. Correlation analysis between growth and physiological indexes of *P. chinense* Schneid seedlings under exogenous GA_3 treatment. (a) Correlation network diagram between growth and physiological indexes. Red lines represent positive correlation, blue lines represent negative correlation, and thicker lines represent significant correlation. (b) Random forest analysis of physiological indicators on the growth of *P. chinense* Schneid seedling. PH: plant height, GD: ground diameter, SFW: stem fresh weight, AFW: aboveground fresh weight, TC: total chlorophyll, SP: soluble protein, SS: soluble sugar.

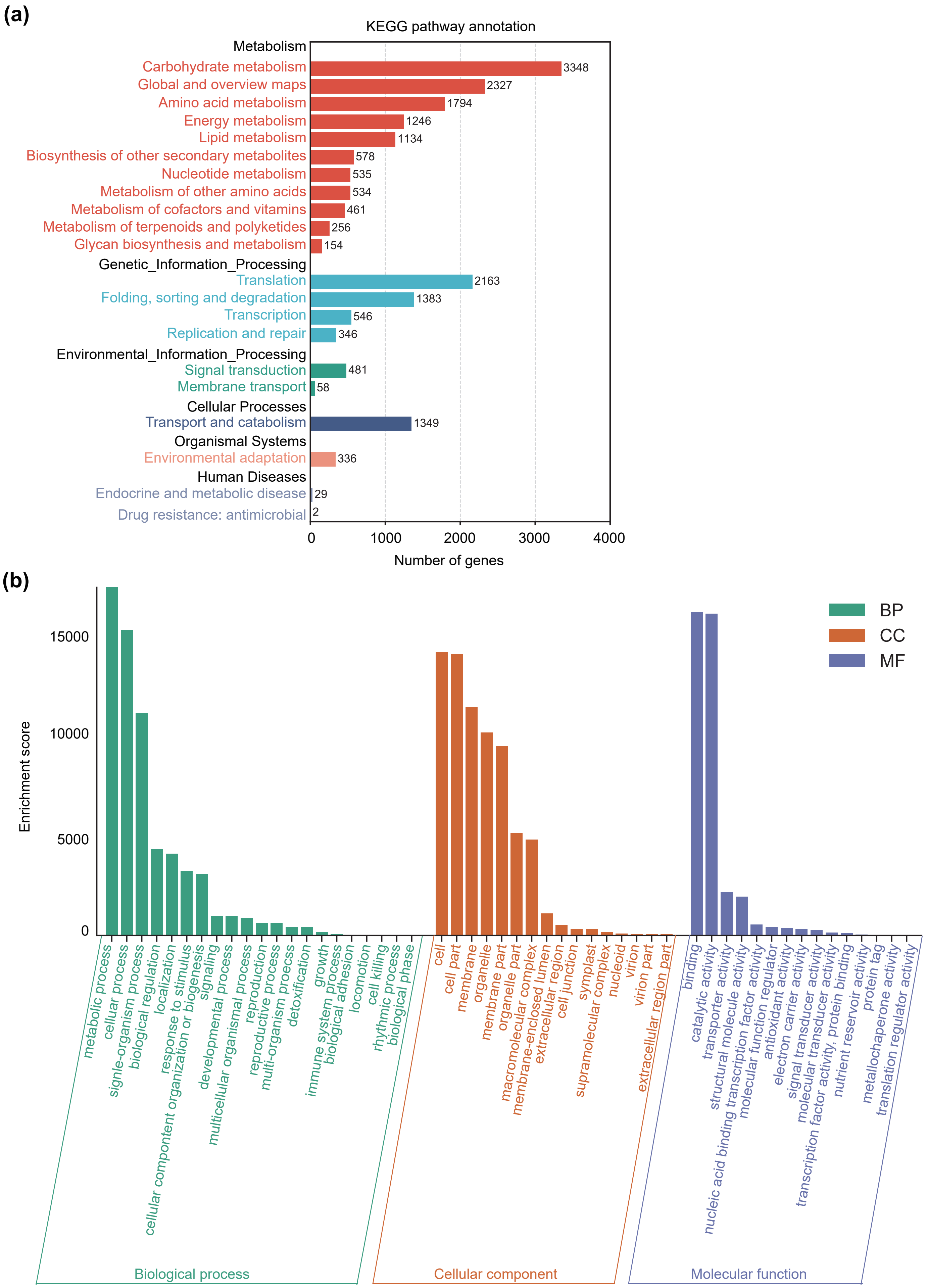

Figure S3. Transcriptome analysis of leaves in *P. chinense* Schneid seedlings under GA_3 treatment. (a) GO annotation of all unigenes. (b) KEGG annotation of all unigenes.

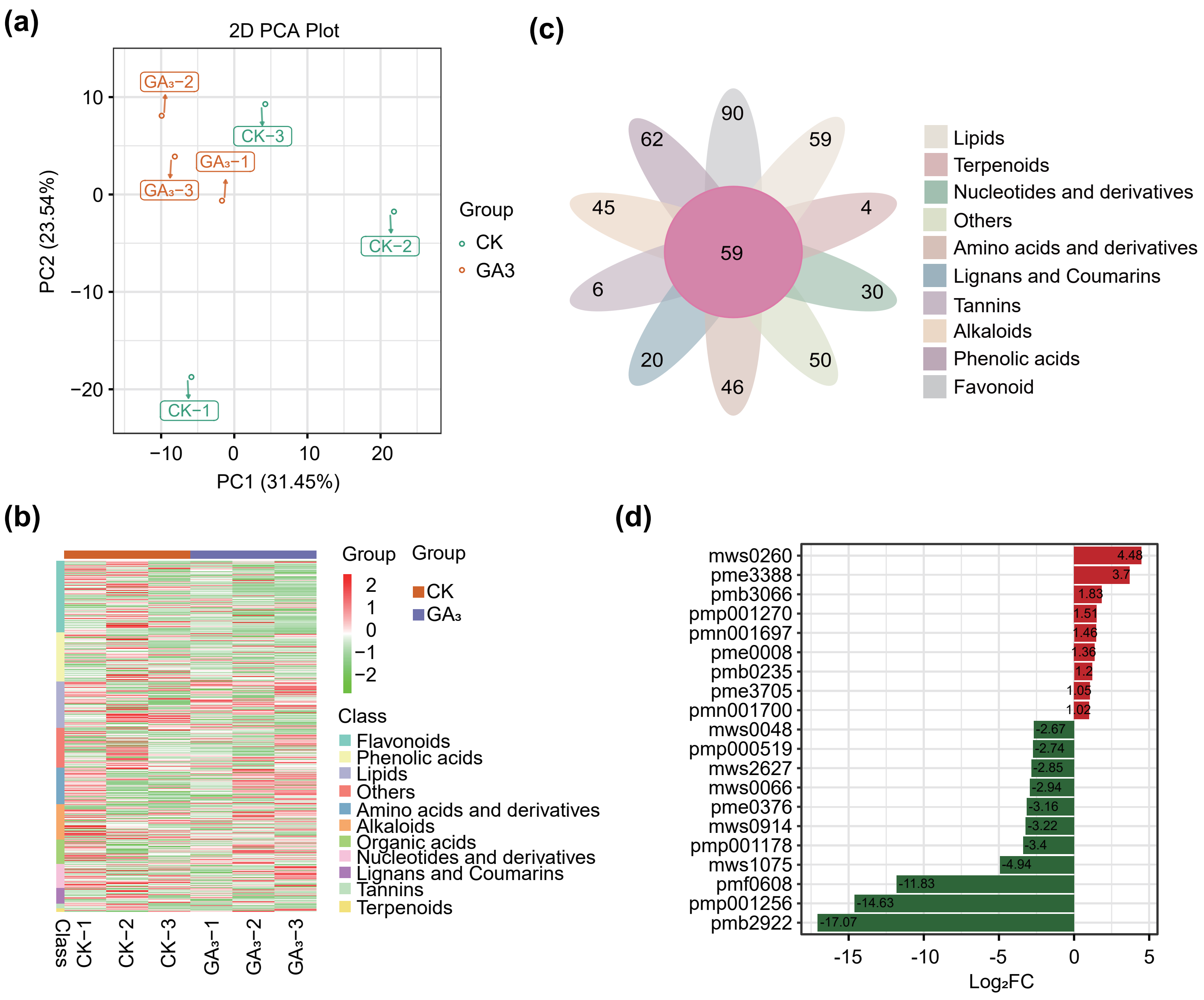

Figure S4. Analysis of metabolome in stem barks of *P. chinense* Schneid seedlings under exogenous GA_3 treatment. (a) PCA analysis. (b) Clustering heat map of metabolite content in the CK group and the GA_3 treatment group. (c) Classification of all metabolites. (d) Bar graph of the top 20 metabolites.

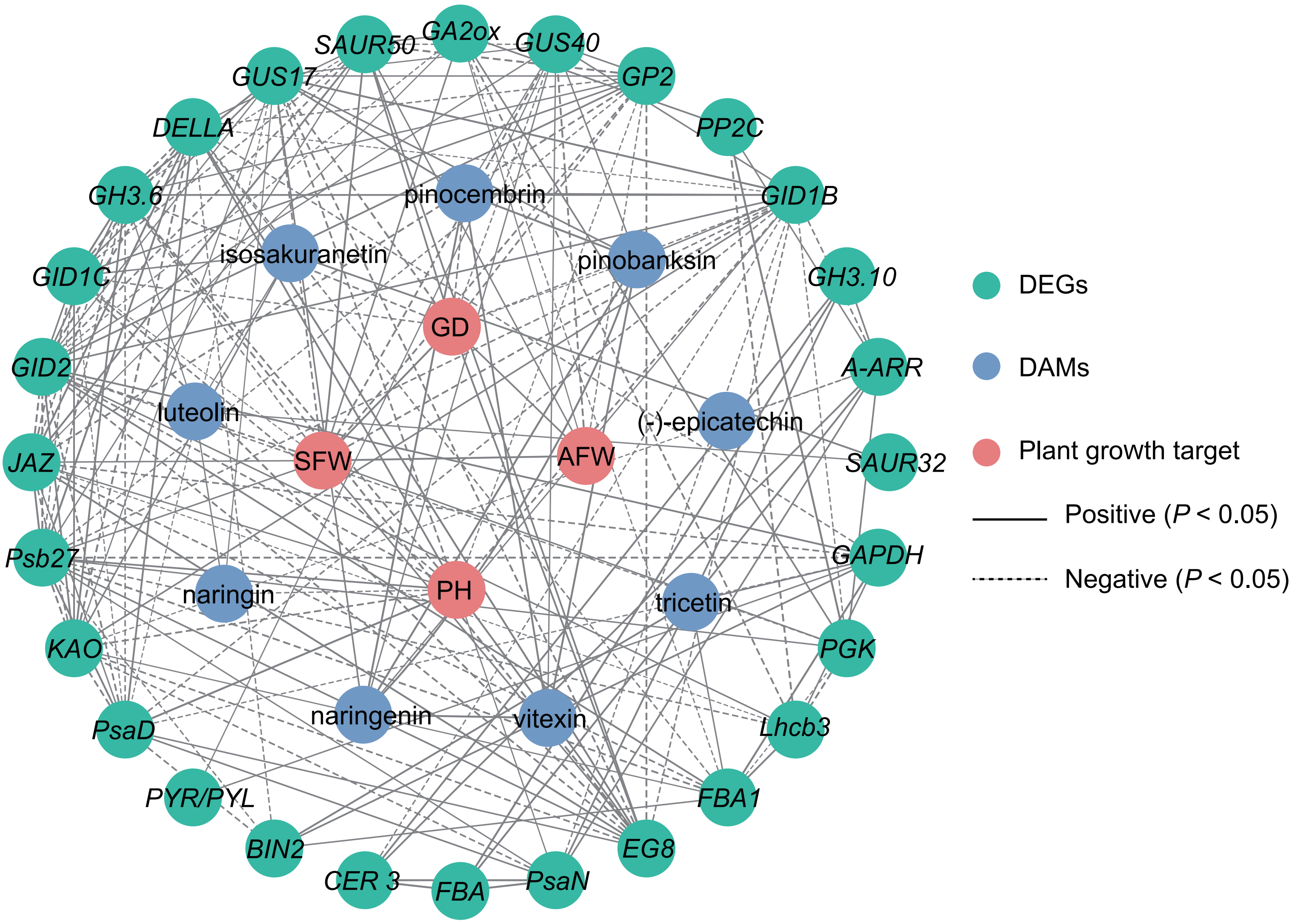

Figure S5. Correlation analysis between DEGs, DAMs and growth indexes of *P. chinense* Schneid seedlings under exogenous GA_3 treatment. TC: total chlorophyll, SP: soluble protein, SS: soluble sugar, PH: plant height, GD: grounddiameter, SFW: stem fresh weight, AFW: aboveground fresh weight.
